# Supplementary material for: Lipid bodies containing oxidatively truncated lipids block antigen cross-presentation by dendritic cells in cancer
Source: Nat Commun. 2017 Dec 14;8:2122. doi: 10.1038/s41467-017-02186-9 (PMC5730553; doi:10.1038/s41467-017-02186-9)
Supplement: Supplementary file 1 — Supplementary Information [file 41467_2017_2186_MOESM1_ESM.pdf]

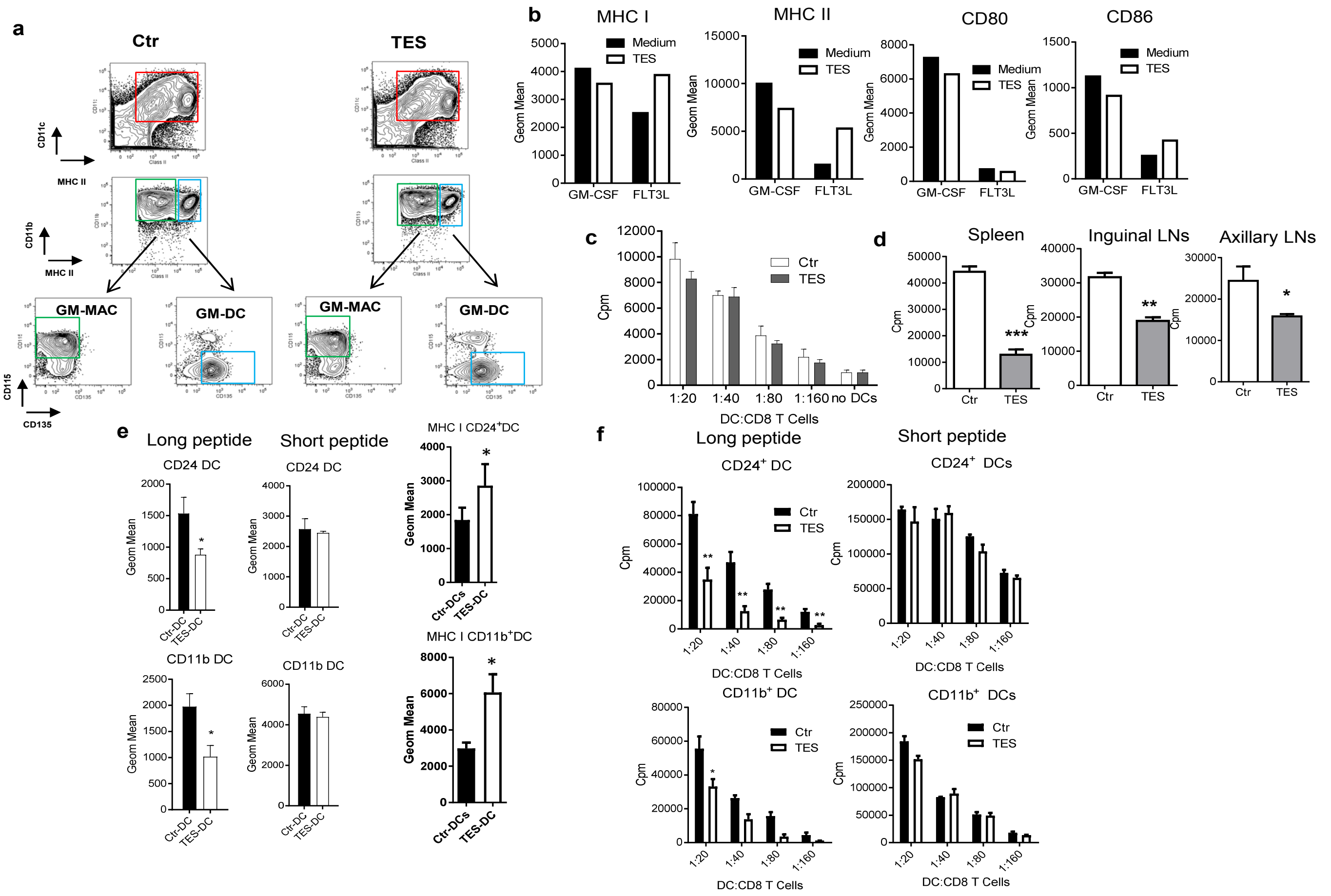

Supplementary Figure 1. **Characterization of DC used in the study. a.** DC were generated from HPC in the presence of GM-CSF for 5 days followed by 48 h treatment with TES. Phenotype was analyzed by flow cytometry. **b.** Expression of indicated surface molecules in DC generated for 6 days in the presence of FLT3L for 6 days and treated for 48 h with TES. **c.** Effect of TES on the ability of DCs to stimulate allogenic T cells. DCs were generated as described above and cultured for 48 h with TES followed by co-culture with allogenic T cells (Balb/c mice) at indicated ratios. Cell proliferation was measured using ^{3}H-thymidine uptake. **d.** Proliferation of OT1 T cells isolated from indicated tissues 7 days after last immunization of mice with DC treated with TES and then loaded with OVA long peptide. Splenocytes and LN cells were stimulated *in vitro* with SIINFEKL peptide and proliferation was assessed in triplicates by ^{3}[H]-thymidine incorporation. Three experiments with the same results were performed. *-p<0.05, **-p<0.01, ***-p<0.001 between mice immunized with control and TES treated DC. **e,f.** Cross-presentation in FLT3L generated DCs. **e.** pMHC **f.** cells proliferation. Bars represent standard deviation (SD). Statistical analysis by unpaired 2-tailed Student t-test with significance determined at *p<0.05 and **P<0.01.

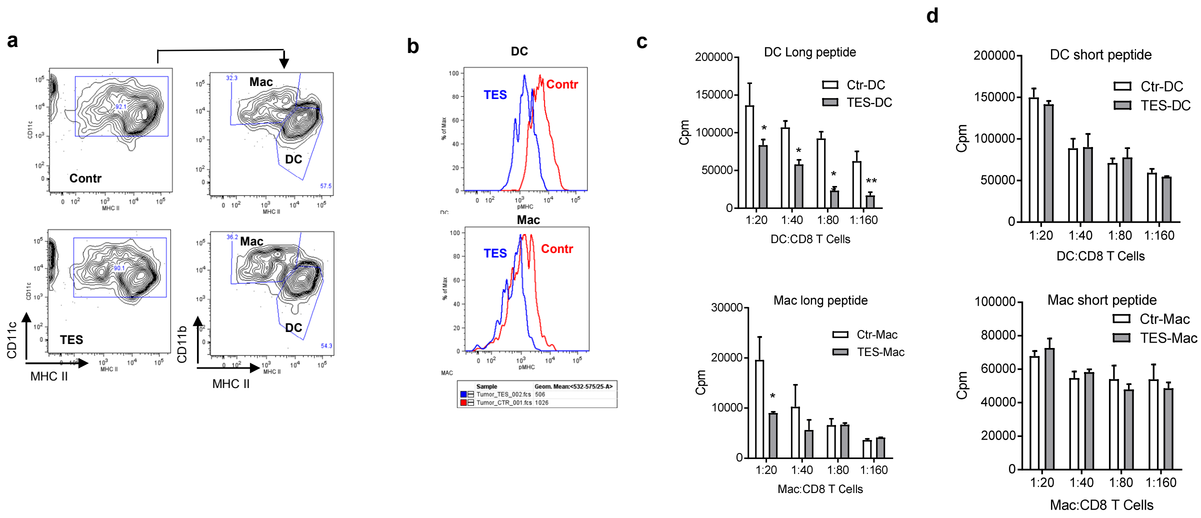

Supplementary Figure 2. Effect of TES on cross-presentation by macrophages and DCs. DCs and macrophages (Mac) were generated from bone marrow HPC for 5 days with GM-CSF and then treated with TES for 48 hours. Cells were loaded with OVA derived long or short peptides and used in the experiments. a. Gating of DCs and Mac. b. Expression of pMHC on the surface of DCs and Mac. Typical example of three performed experiments is shown. c. Proliferation of OT-1 CD8+ transgenic T cells stimulated with DCs or Mac loaded with OVA-derived long peptide. Proliferation was measured in triplicates. Three experiments were performed. * - $p<0.05$; ** - $p<0.01$ from control. d. Proliferation of OT-1 CD8+ transgenic T cells stimulated with DCs or Mac loaded with OVA-derived short peptide. Three experiments were performed.

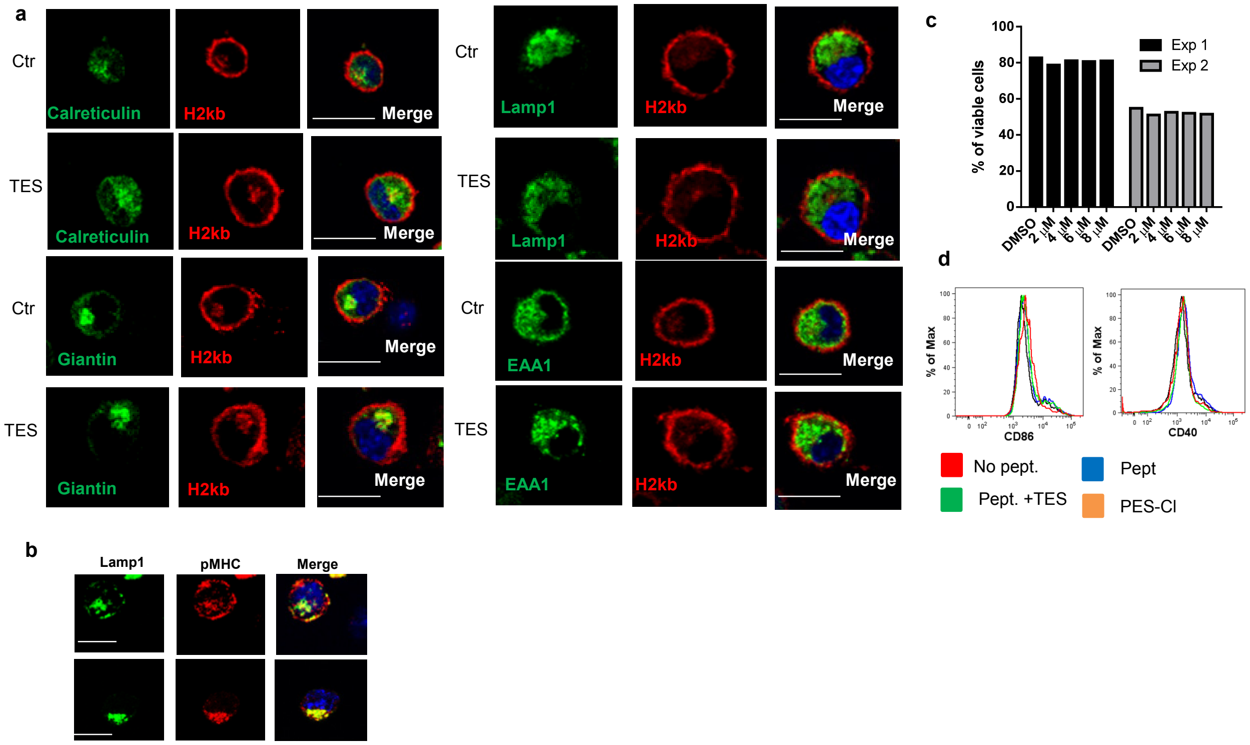

Supplementary Figure 3. **Distribution of MHC I (H2K^{b}) in DC treated with TES. a.** DC were generated from HPC in presence of GM-CSF and treated with TES for 48 hours. Distribution of H2K^{b} was analyzed in different intracellular compartments after loading with long OVA-derived peptides by confocal microscopy. Calreticulin – ER, EEA1 – early endosomes, LAMP1 – late endosomes/lysosomes, giantin-Golgi. Scale bars = 10 µm **b.** CD103^{+}DCs were generated *in vitro* from bone marrow progenitors using combination of GM-CSF and FLT3L and cultured with TES for 48h. Confocal microscopy of sorted CD103^{+} DCs loaded with long peptide. Scale bars = 50 µm; **c.** DC viability after treatment with HSP70 inhibitor PES-Cl. **d.** Expression of co-stimulatory molecules in DCs treated with PES-Cl. Bars represent standard deviation (SD). Statistical analysis by unpaired 2-tailed Student t-test with significance determined at *p<0.05 and **P<0.01.

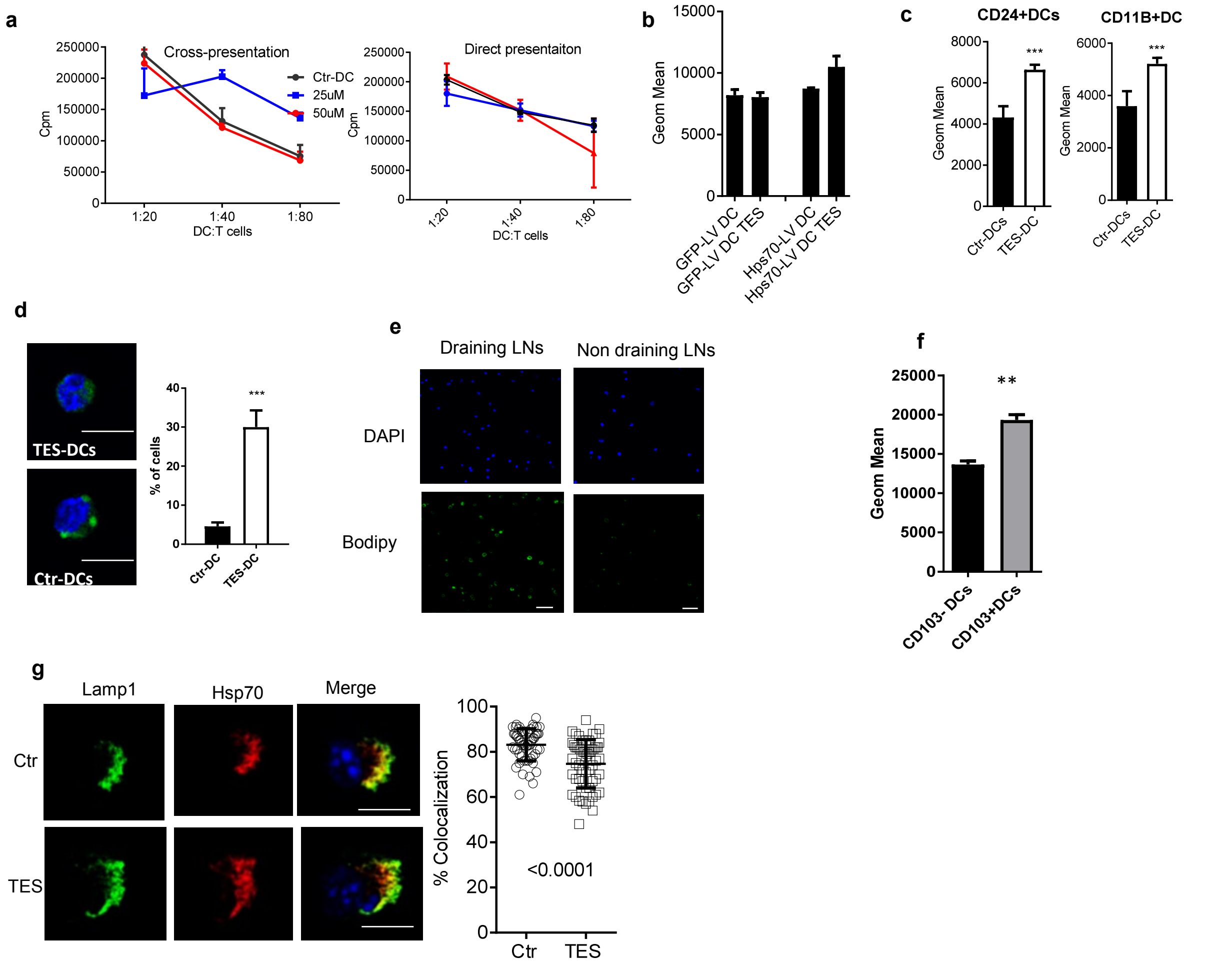

Supplementary Figure. 4. Accumulation of lipids in DCs. **a.** Effect of HSP90 inhibitor 17AAG on the ability of DCs to cross-present antigens. DCs were generated from BM as described in methods and were treated with indicated concentrations of 17AAG during 24 hr loading with long OVA-derived peptide. Cells were then washed and used for stimulation of OT1 $CD8^{+}$ T cells. Two experiments with the same results were performed. **b.** Effect of transduction of DCs with control and Hsp70 lentivirus on expression of MHC class I. **c.** DCs were generated with FLT3L and treated with TES for 48 hrs. Amount of lipids (bodipy fluorescence) was measured in different populations of DCs, by using bodipy and analyzed by flow citometry *** $p<0.001$, n=5. **d.** Confocal microscopy of lipid content in DC generated with FLT3L and treated with TES. The proportion of DCs containing more than 3 large LB was quantified from DCs $p<0.001$, n=5. **e.** Confocal microscopy of lipid content in DC sorted from draining or non-draining LN of LLC-bearing mice. Scale bar = 10 µm. F. Lipid contents in CD103+ and $CD103^{-}$ DCs isolated from dLNs of tumor bearing mice. Bars represent standard

deviation (SD). Statistical analysis by unpaired 2-tailed Student t-test with significance determined at *p<0.05 and **P<0.01. **g.** Co-localization of HSP70 and Lamp1 in BM derived DC treated with TES. Left –typical example of staining. Scale bar = 10 μm. Right – % of co-localization for HSP70 and Lamp1 in each DCs calculated by using Leica Software. At least 40 cells were counted in each sample, total 4 samples. Bars represent standard deviation (SD). Statistical analysis by unpaired 2-tailed Student t-test with significance determined at *p<0.05 and **P<0.01.

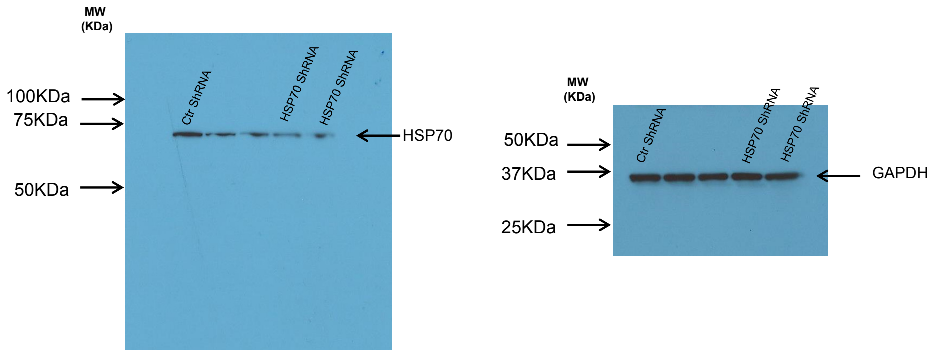

Supplementary Figure 5. Raw data on HSP70 expression after transduction of DCs with Hsp70 shRNA. DCs were transduced with lentiviruses containing wither control or Hsp70 shRNA. Raw data of WB are shown.

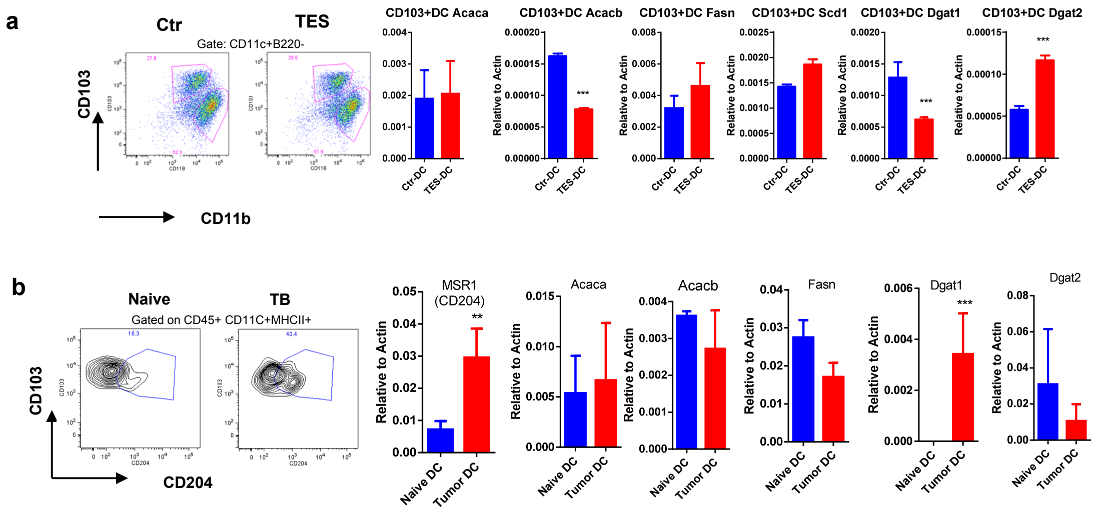

Supplementary Figure 6. Expression of components of lipogenesis in DCs. a. $CD103^{+}$ DCs generated ex vivo for 7 days using GM-CSF and FLT3-L. **b.** CD103+ DCs sorted from lymph nodes of naïve and LLC TB mice (3 weeks after tumor inoculation). Indicated gene expression was evaluated by RT-qPCR and normalized to β-actin. Each group included 3 mice. *** - p <0.001. Acetyl-CoA-carboxylase 1 and 2 (Acc1,2, encoded by *acaca* and *acacb*), fatty acid synthase (*fasn*), stearoyl-CoA-desaturase (*scd1*), acyl-CoA:diacylglycerol acyltransferases (*dgat1, dgat2*).

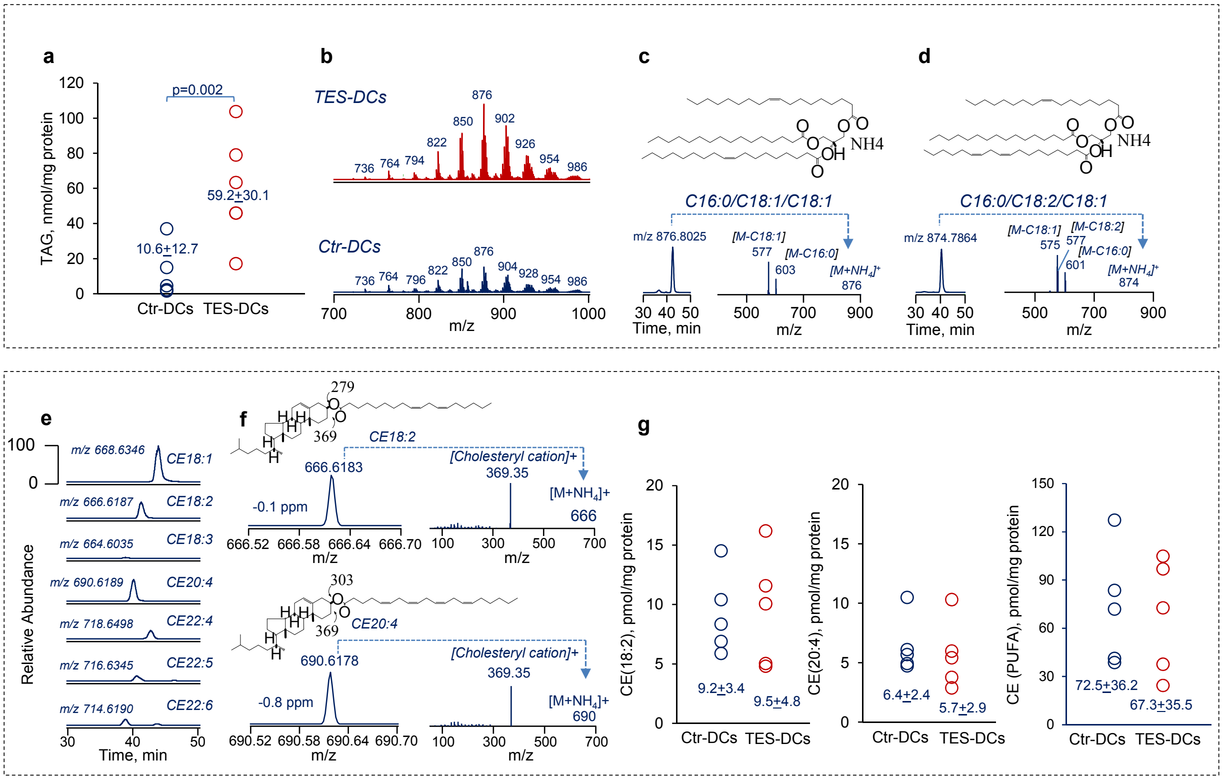

Supplementary Figure 7. LS-MS analysis of lipids in DC treated with TES. a. Total amount of TAG in control DC and DC treated with TES. **b**. Typical LC-MS spectra of TAG in control DC and DC treated with TES. **c,d.** LC-MS profiles and LC-MS/MS spectra confirming structural analysis of ions at m/z 876 and 874 for TAG molecular species, containing C16:0/C18:1/C18:1 and C16:0/C18:2/C18:1, respectively. Possible structures are inserted. **e-g.** The amount of cholesterol esters in DC treated with TES. **e**. Typical LC-MS profiles of major CE molecular species, containing PUFA. **f**. LC-MS1 and LC-MS/MS spectra confirming structural analysis of ions at m/z 666 and 690 for CE molecular species, containing CE 18:2 and CE 20:4, respectively. Possible structures are inserted. **g.** Content of individual molecular species of CE 18:2, CE 20:4 and total amount of CE, containing (PUFA) in control DC and DC treated with TES.

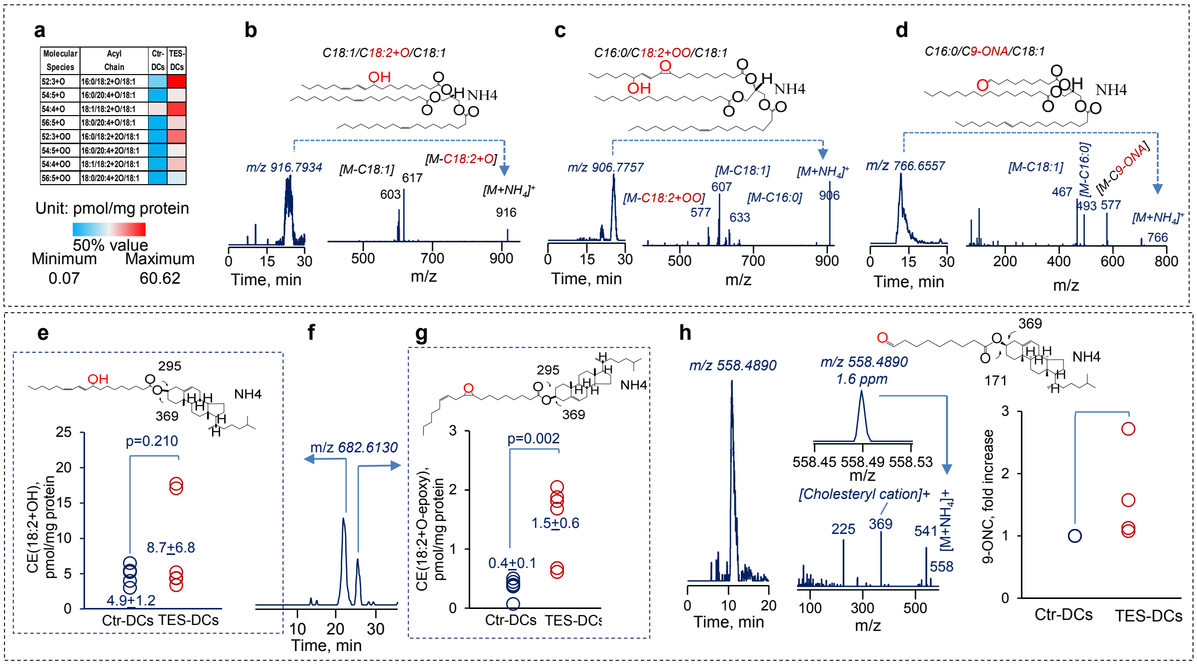

Supplementary Figure 8. LS-MS analysis of oxidized TAG in DC treated with TES. a. The heatmap of singly- and doubly-oxygenated TAG molecular species in control DC and DC treated with TES. **b-d.** LC-MS profiles and LC-MS/MS spectra confirming structural analysis of ions at m/z 916, 906 and 766 for mono-, di-oxygenated and oxidatively truncated TAG molecular species, containing C18:1/C18:2+O/C18:1, and C16:0/C18:2+2O/C18:1 and C16:0/C9-ONA/C18:1, respectively. Possible structures are inserted. **e-h.** Typical LC-MS profile (**f**) and content of oxygenated CE 18:2+O molecular species, containing hydroxy- (**e**) and epoxy- (**g**) groups. Typical LC-MS profile (**h**) and LC-MS1, LC-MS/MS and fold increase of oxidatively truncated CE 9-ONA detected in TES treated DC. Possible structure is inserted.

a

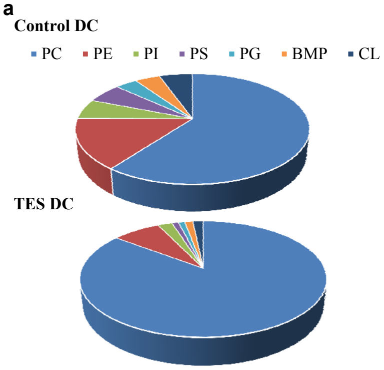

b

Molecular Species	m/z	Ctr DC	TES DC
PC(30:0)	764.5439		
PC(32:2)	788.5451		
PC(32:1)	790.5602		
PC(32:0)	792.5759		
PCp(34:0)	804.6135		
PC(34:2)	816.5759		
PC(34:1)	818.5918		
PC(34:0)	820.6092		
PCp(36:3)	826.5970		
PCp(36:1)	830.6266		
PC(36:5)	838.5601		
PC(36:4)	840.5754		
PC(36:3)	842.5920		
PC(36:2)	844.6066		
PC(36:1)	846.6226		
PC(36:0)	848.6378		
PCp(38:5)	850.5957		
PCp(38:4)	852.6122		
PC(38:6)	864.5753		
PC(38:5)	866.5914		
PC(38:4)	868.6073		
PC(36:3)+2O	874.5781		
PC(38:4)+1O	884.6025		
PC(38:3)+1O	886.6225		
PC(38:2)+1O	888.6292		
PC(40:6)	892.6095		
PC(40:5)	894.6217		
PC(38:4)+2O	900.5940		

Unit: pmol/1*10[6] cells

Minimum 0.04 | 50% value | Maximum 74.82

c

Acyl chains	m/z	Ctr DC	TES DC
16:0/18:2-OH/18:1	912.7651		
16:0/9-ONA/18:1	766.6557		
16:0/HOOA/18:2	766.6202		
16:0/9-ONA/18:0	768.6713		
18:0/9-ONA/18:1	794.6870		

Unit: pmol/1*10[6] cells

Minimum 0.36 | 50% value | Maximum 103.92

Supplementary Figure 9. Redox phospholipidomics of LB in DCs. DCs were generated *ex vivo* from bone marrow progenitors using GM-CSF and FLT-3L and treated with TES for 48 hrs. LB were isolated using gradient centrifugation. a. Distribution of phospholipid classes in lipid bodies, b. heat map of individual PC molecular species in LB from control and TES DC. PC – Phosphatidylcholine, PE – Phosphatidylethanolamine; PS - Phosphatidylserine ; PI – Phosphatidylinositol; CL – cardiolipin, PG – phosphoglycerides, BMP – bis(monoacyulglycero)phosphate; **c.** Heat-map of oxidatively-truncated TAG species and hydroxy-TAG species in LD from control and TES DC.

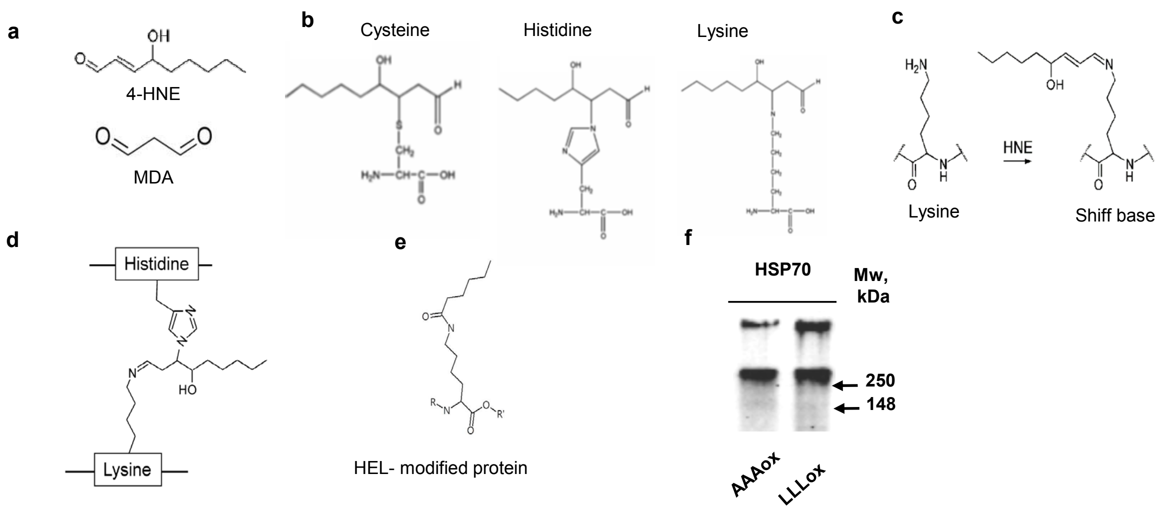

Supplementary Figure 10. Oxidative modifications in fatty acids. a. The structure of 4-hydroxy-2-nonenal (4-HNE) and malonyl-dialdehyde (MDA). **b.** The structure of cysteine, histidine and lysine with depicted nucleophilic thiol (−SH) or amino (−NH2) groups. **c.** Schiff base formation by HNE with lysine. **d.** Formation of pyrrole formation **e.** Modification of proteins by bifunctional lipid peroxidation products. **f.** Detection of HEL- adducts formed after incubation of HSP70 with arachidonic and linoleic acids containing TAG/DOPC oxidized by MPO.

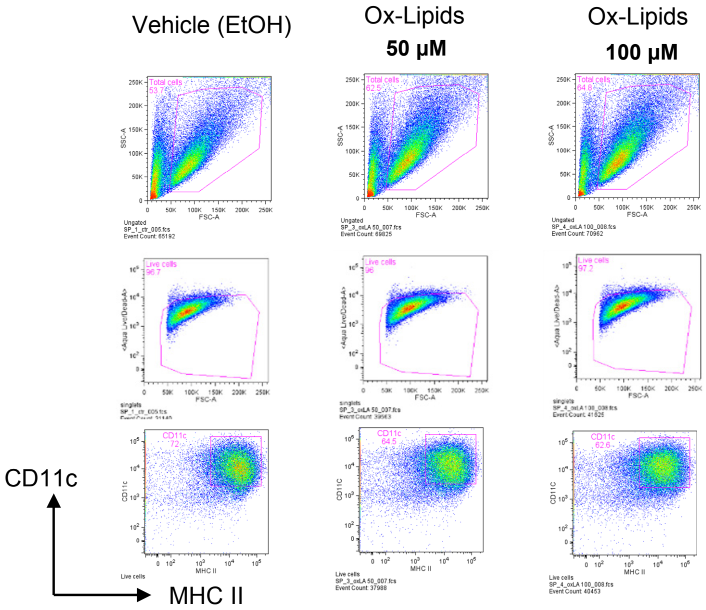

Supplementary Figure 11. Effect of oxLA on DC phenotype. DC were generated from HPC in presence of GM-CSF and treated with different amounts of ox-LA or vehicle (EtOH) for 4 hours in serum free medium. The phenotype was analyzed by flow cytometry. Typical example of three experiments is shown.

Supplementary Table 1. Major oxygenated metabolites of LA

Name, ID	[M-H], m/z	ppm	Formula	Integrated Intensity	Possible structure
4-hydroxy-2E-nonenal	335.1367*	3.6ppm	$C_9H_{16}O_2$	2.37E+05	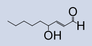
6-oxo-heptanoic acid	143.0702	0.3 ppm	$C_7H_{12}O_3$	2.19E+07	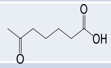
7-oxo-octanoic acid	157.0853	2.4 ppm	$C_8H_{14}O_3$	2.72E+08	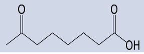
8-hydroxy-octanoic acid	159.1018	1.6 ppm	$C_8H_{16}O_3$	4.66E+08	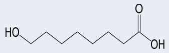
9-oxo-nonanoic acid	171.1020	2.3 ppm	$C_9H_{16}O_3$	2.87E+09	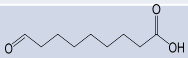
Azelaic acid	187.0970	2.8 ppm	$C_{92}H_{16}O_4$	1.32E+09	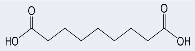
12-oxo-9-dodecenoic acid	211.1337	3.9 ppm	$C_{12}H_{20}O_3$	2.17E+09	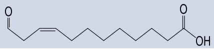
9-Hydroxy-12-oxo-10-dodecenoic acid	227.1289	4.6 ppm	$C_{12}H_{20}O_4$	5.34E+08	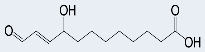
13-keto-9,11-octadecadienoic acid	293.2119	2.8 ppm	$C_{18}H_{30}O_3$	8.83E+10	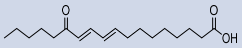
13-hydroxy-9,11-octadecadienoic acid	295.2260	2.5 ppm	$C_{18}H_{32}O_3$	9.10E+08	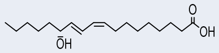
9-hydroperoxy-10,12-octadecadienoic acid	311.2229	3.9 ppm	$C_{18}H_{32}O_4$	1.81E+10	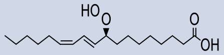
9,10-epoxy-13-hydroxy-11-octadecenoic acid	311.2229	3.9 ppm	$C_{18}H_{32}O_4$	5.12E+10	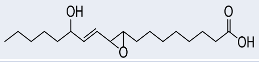
7,10-dihydroxy-8-octadecenoic acid	313.2383	3.6 ppm	$C_{18}H_{30}O_5$	1.47E+08	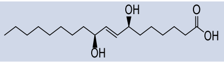
12,13-epoxy-11-hydroxy-8-oxo-9-octadecenoic acid	325.2024	4.4 ppm	$C_{18}H_{30}O_5$	4.06E+07	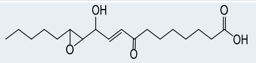

A

LA oxygenated products, containing aldehyde groups were detected after derivatization with dinitrophenylhydrazine (DNPH). Derivatized products were analyzed by LC-ESI/MS/MS in the negative-ion mode and parent ions and characteristic fragments for each aldehyde derivative were detected. For example, HNE–DNPH-HNE has a parent ion at m/z 335.1367 (3.6 ppm) and fragments at m/z 152, 163, 167, and 182, respectively. DNPH-9-ONA has a parent ion at m/z 351.1315 (2.8 ppm) and major fragments at m/z 152 and 163, respectively.
